# Identifying unknown Indian wolves by their distinctive howls: its potential as a non-invasive survey method

**DOI:** 10.1038/s41598-021-86718-w

**Published:** 2021-03-31

**Authors:** Sougata Sadhukhan, Holly Root-Gutteridge, Bilal Habib

**Affiliations:** 1 Animal Ecology and Conservation Biology, Wildlife Institute of India, Dehradun, 248001 India; 2 Animal Behaviour, Cognition and Welfare Group, University of Lincoln, Lincoln, UK; 3 Reby Lab, School of Psychology, University of Sussex, Brighton, UK

**Keywords:** Environmental sciences, Ecology, Behavioural ecology, Conservation biology, Ecological modelling, Population dynamics, Zoology, Animal behaviour

## Abstract

Previous studies have posited the use of acoustics-based surveys to monitor population size and estimate their density. However, decreasing the bias in population estimations, such as by using Capture–Mark–Recapture, requires the identification of individuals using supervised classification methods, especially for sparsely populated species like the wolf which may otherwise be counted repeatedly. The cryptic behaviour of Indian wolf (*Canis lupus pallipes*) poses serious challenges to survey efforts, and thus, there is no reliable estimate of their population despite a prominent role in the ecosystem. Like other wolves, Indian wolves produce howls that can be detected over distances of more than 6 km, making them ideal candidates for acoustic surveys. Here, we explore the use of a supervised classifier to identify unknown individuals. We trained a supervised Agglomerative Nesting hierarchical clustering (AGNES) model using 49 howls from five Indian wolves and achieved 98% individual identification accuracy. We tested our model’s predictive power using 20 novel howls from a further four individuals (test dataset) and resulted in 75% accuracy in classifying howls to individuals. The model can reduce bias in population estimations using Capture-Mark-Recapture and track individual wolves non-invasively by their howls. This has potential for studies of wolves’ territory use, pack composition, and reproductive behaviour. Our method can potentially be adapted for other species with individually distinctive vocalisations, representing an advanced tool for individual-level monitoring.

## Introduction

Accurate population estimates are a critical part of wildlife biology, conservation and inform management strategies^1^. Informed management decisions rely on accurate estimates which can be hard to achieve but are critical as the conservation status of any species is dependent on its population size, which is inversely correlated with extinction risk^2^. Therefore, it is of the foremost importance to have a statistically robust population estimation technique. However, widely used population estimation methods such as camera trapping and sighting-based distance sampling fall short in analysing population trends of certain elusive species or species living in extensive home ranges^3,4^. Many of these species are vocally active, which inspired scientists to study the effectiveness of an acoustics-based population abundance model for these species^5–8^. Acoustic monitoring has long been used to monitor the presence of aquatic animals, amphibians, insects, and birds^9–13^. The critical advantages of acoustic monitoring are that it can be operative at day and night^14^ and detect visually cryptic species or those spread over large home ranges^7,15,16^. Like camera traps, passive acoustics devices can operate throughout the day for weeks without intervention, and this perpetual data can be analysed easily with the advancement of methodologies for automating the process^17^. Recordings from these devices can be used in calculating a wide range of metrics including acoustic indices^18,19^, animal diversity^19,20^, animal localisation^21–23^, and density^24,25^ estimation. This density estimation is mostly obtained through Spatially Explicit Capture-Recapture (SECR) that relies on multiple recording stations for Capture-Mark-Recapture (CMR), and instead of ‘recapture’ with time, it considers ‘redetection’ in different points in space^24–26^. This methodology is applied to individuals that are not identifiable from their calls^25,27^. The conventional CMR model requires identification at the individual level^27,28^, but it provides a robust population estimation^28^ and much additional information such as home-range, survival rate, animal movement pattern, and population viability analysis^29^. However, the difficulty of successfully identifying unknown individuals from their calls has prevented its use for more species, though new techniques are being developed for some species, including the use of unsupervised classifiers to cluster calls^30^. Here, we explore the potential of identifying individuals through supervised classification from their vocal features to potentially improve identification to the point where CMR surveys are possible for an elusive and wide-ranging species.

Like other grey wolf subspecies, Indian grey wolves are known for their long-ranging communication via howls^31^. Howling is a social communication process, vital for the overall behaviour of many canid species^32^. It has evolved in wolves to communicate with other group members over a long distance as well as to demarcate their vast territories^33^. Due to its high amplitude and low frequency, a howl can travel for six kilometres or more^34–36^. Hence, an acoustics survey using howling may be more advantageous than a visual survey or other methods, such as snow-tracking^22,23,35,37^. As vocalisations of wolves were found to be highly variable within and among individuals^31,38^, the howl is a useful tool to identify individuals^39–41^; thus, wolves are ideal candidates for acoustic monitoring techniques.

Previously the ‘*Howlbox*’, a self-contained active acoustics-monitoring device that broadcasts howls and records the responses automatically, was tested for its capability to detect wolves^42,43^. This device was unsuccessful in surveying wolves due to low detection rate as, instead of howling back, the wolves visited the device site without howling, and various technical failures^42^. A few studies using passive acoustic devices show the potentiality of successful localisation and monitoring of the grey wolf^23,44^. However, these only allowed for presence to be detected and stopped short of individual identification. In contrast, the identification of wolves from their distinctive howls will open an opportunity for more conventional CMR methods^45^, and this will improve population estimation without bias and help to measure other ecological variables, such as site occupancy and home-range. With the ability to identify individual wolves from howl recordings, information on population sizes, dispersal patterns, pack composition and the presence of pups could be obtained. These would be used to develop conservation management strategies and to examine population trends with howl surveys conducted over multiple years. Therefore, our study aimed to record howls from Indian wolves (*Canis lupus pallipes*) and test the feasibility of identifying unknown individuals from their howls alone using a supervised classification method.

## Methods

### Study species

Indian wolf, subspecies of the grey wolf is among the keystone species found in the Central Indian landscape^46^ and reside in arid grasslands, floodplains, and the buffer of dense forests^46–49^. The Indian wolf plays a significant ecological role in controlling ungulate populations in the human-dominated landscapes^50,51^. The population status of Indian wolves is entirely unknown^52^. It is known that Indian wolves face a major threat from humans as their habitat is increasingly used by humans, and human-wildlife conflict is increasing^53^. Therefore, time is a critical factor to their conservation. The major challenges for population estimation of the wolf are its vast home range of ~ 230 km^2^^48^ and that they actively avoid camera traps because of camera sound, light, and odour emission^54^. Since implementing standard population monitoring tools in these landscapes is a tremendous challenge, monitoring their population through howls can be an essential technique. The average fundamental frequency and duration of Indian wolf howls are 422 Hz and 5.21 s, respectively^55^. Due to its low-frequency range and longer duration, it can be heard from an extended distance like howls of other subspecies^23,35,36^.

### Study site

The study was conducted on captive individuals of Jaipur Zoo and free-ranging, wild wolves of Maharashtra, India.

Jaipur Zoo is situated at the heart of Jaipur City, Rajasthan, India. Since Jaipur is one of the major tourist destination and capital of Rajasthan, the anthropogenic noise is reasonably high in and around the zoo. All the wolves (n = 10) in Jaipur zoo were offspring of captive-bred individuals except one adult male recently captured from a wild population of Rajasthan.

**Figure 1:**
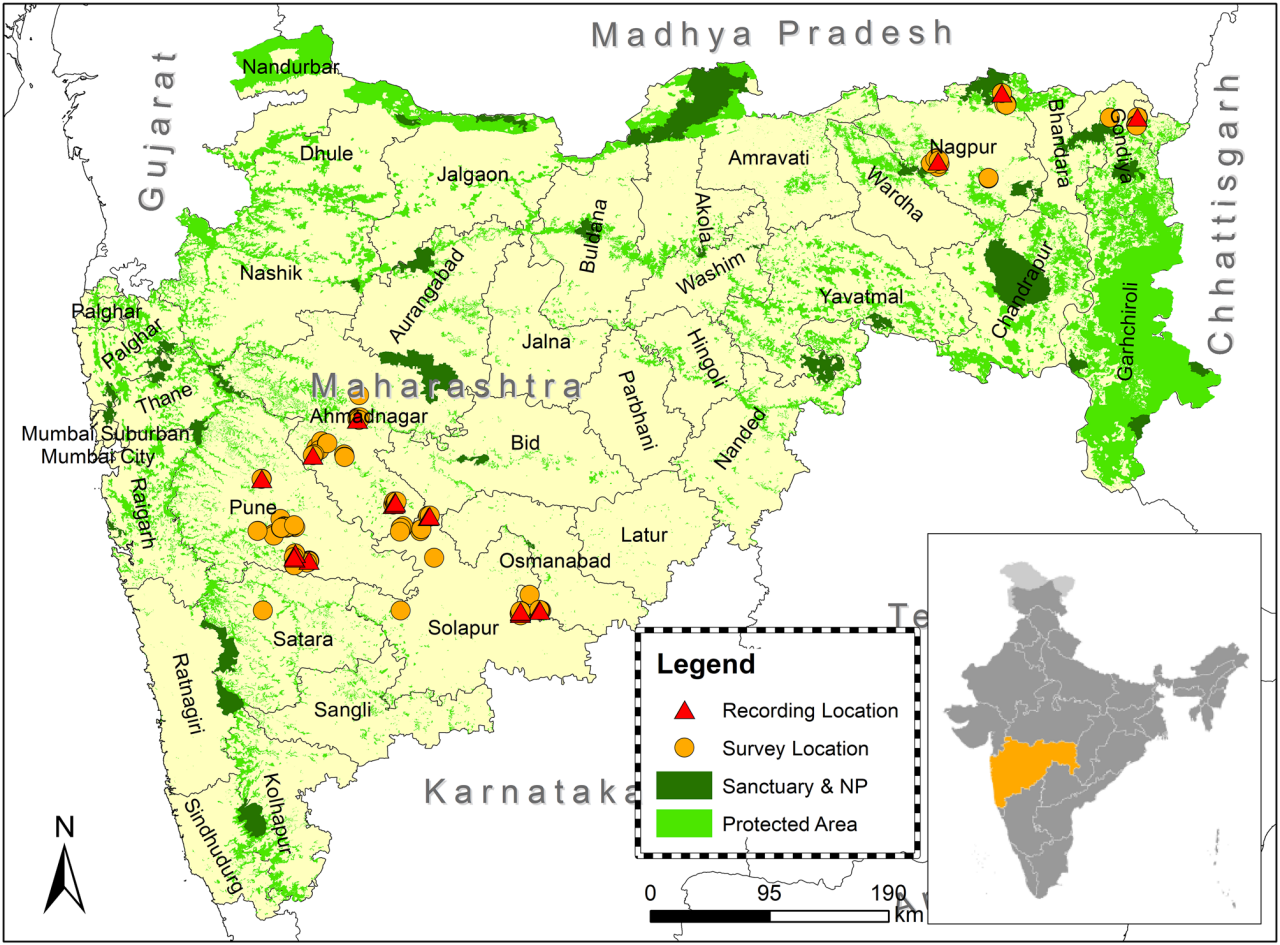
Map showing howling recording locations of the free-ranging wolf in six districts of Maharashtra. Yellow round bullets indicate the survey locations and Red triangular bullets represent the howling recording sites.

The data of free-ranging wild wolves were collected from six districts of Maharashtra. Pune, Ahmednagar, Solapur and Osmanabad (Fig. 1) districts fall under the semi-arid drought-prone area of the Deccan peninsula Biogeographic Zone (Zone 6)^56^. The dominant habitat type in our sampling areas was *Deccan thorn scrub forests*^57^. The terrain is gently undulating with mild slopes and flat-topped hillocks with intermittent shallow valleys, which forms the primary drainage channels. Grassland area is distributed in fragmented patches, creating a mosaic of grazing land, agricultural land and human settlements. Striped hyenas (*Hyaena hyaena*), golden jackals (*Canis aureus indicus*), and Indian leopards (*Panthera pardus fusca*) are the co-predators in this landscape^48,58^. Wild prey include blackbucks (*Antilope cervicapra*), chinkaras (*Gazella bennettii*) and wild pigs (*Sus scrofa cristatus*); but a significant part of their diet is domestic livestock^48,50,59^.

In Maharashtra, Nagpur and Gondia districts come under the central Deccan Plateau with Tropical dry deciduous broadleaf forests^56,57^. Due to moderate to high rainfall, vegetation is dense in most of the areas. Our sampling areas were mostly packed with open forest and modest density forest. The terrain is generally flat. Nagpur division is surrounded by Many National parks and Sanctuaries. Wolves are primarily found in the buffer areas of these protected areas. Co-predators in those stretches are tigers (*Panthera tigris tigris*), Indian leopards, sloth bears (*Melursus ursinus*), striped hyenas, dholes (*Cuon alpinus*), and golden jackals. Prey species are sambar (*Rusa unicolor*), nilgai (*Boselaphus tragocamelus*), chital (*Axis axis*), chousingha (*Tetracerus quadricornis*), and wild pigs.

### Data collection

The howls from the Indian wolves were recorded from November 2015 to July 2016. The howls were recorded during the systematic howling surveys accompanied by the opportunistic and spontaneous recordings of captive and free-ranging wolf howls. Howling surveys were done in the early morning (from 4:30 am onwards) and early evening hours (up to 7:45 pm) [time varies depending on sunrise and sunset]. The survey protocol was adapted from Harrington and Mech^60^. Each howling session consisted of five trials with three-minute intervals. A series of 50-s-long pre-recorded solo howls (from an individual in Jaipur Zoo) was played three times with increasing amplitude; the session was followed by a 50-s-long chorus howl (from three individuals in Jaipur Zoo) in the order of mid and highest amplitude level of the speaker respectively. A 40-W JBL Xtreme speaker (Harman International Industries, 2014) was used for the playbacks. If howling responses were recorded, the playback session was terminated and repeated after 15 to 20 min. All howls were recorded in a single microphone setup, using a Blue Yeti Pro USB Condenser Microphone (Blue Microphone, 2011) attached with Zoom H4N Handheld Audio Recorder (Zoom Corporation, 2009) with a sampling rate of 44.1 kHz and 16-bit depth.

### Ethical approval

The study on captive wolves in zoos was done with the permission of the Director of Jaipur Zoo and the Forest Department of Rajasthan, India [Letter no- 3(04)-II/CCFWL/2013/4586–87; Dated 30th Oct 2015]. The survey of free-ranging wolves of Maharashtra was performed with the consent of the Principal Chief Conservator of Maharashtra Forest Department [Letter no- 22(8)/WL/CR-947(14–15)/1052/2015–16; Dated- 6th Aug 2015]. No animal was harmed during the study, and the standard non-invasive protocol of howling survey was maintained. All the data collection were approved by the Animal Ethics committee of Wildlife Institute of India, Dehradun, India.

### Feature extraction

**Figure 2:**
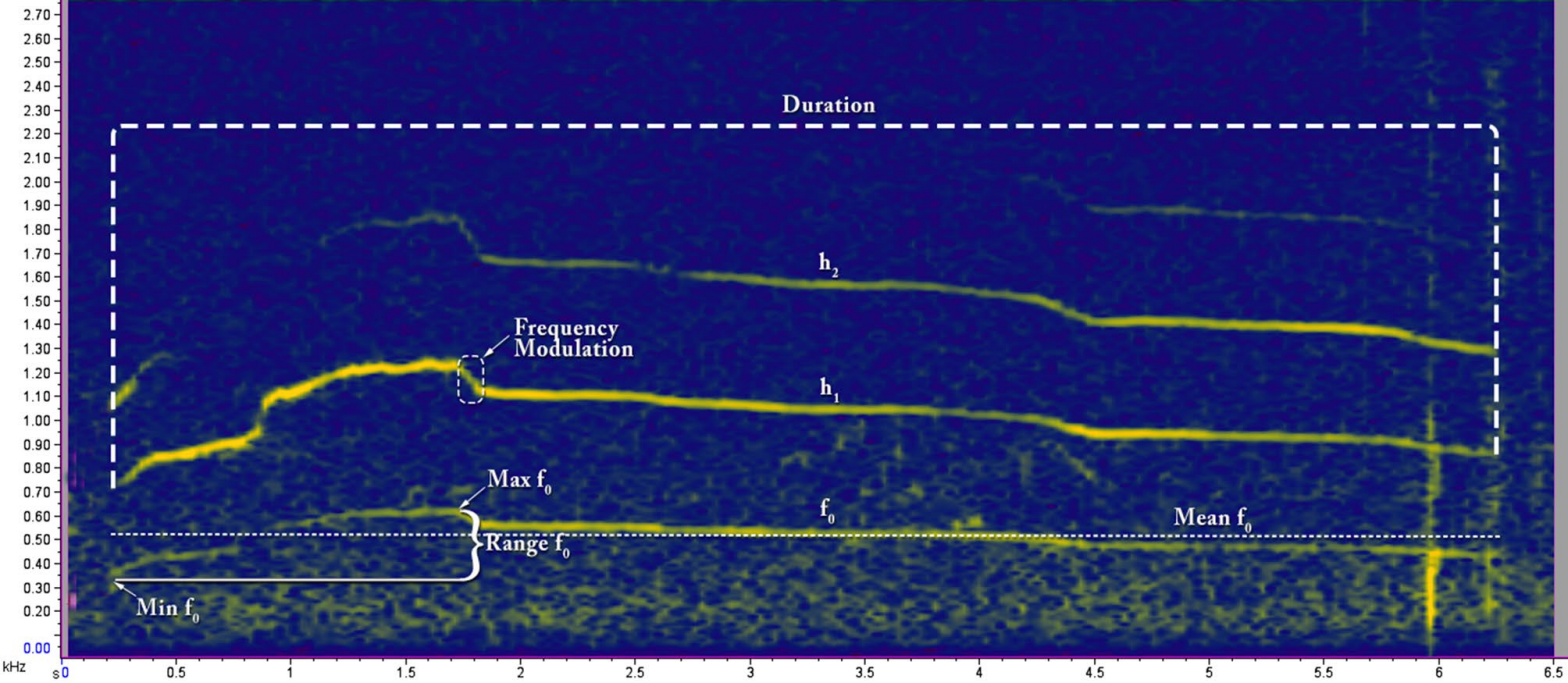
Spectrogram of Gangewadi Wolf howl (160203-001_Gangewadi2_A5) showing how different variables were measured.

**Figure 3:**
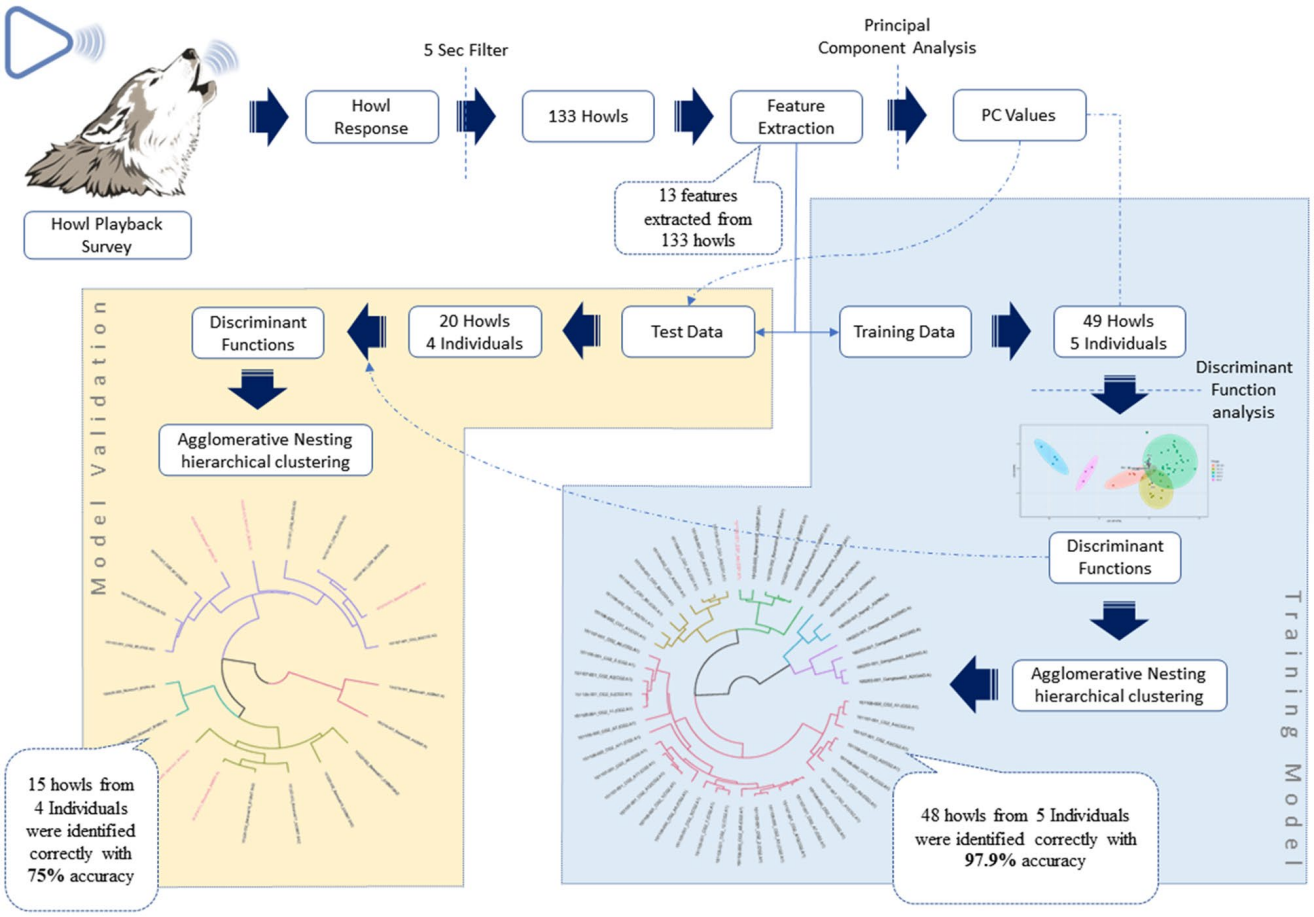
The pictorial representation of methodology for identifying unknown Indian wolves by their howls.

The howls were sorted, and spectrograms were generated using a *Discrete Fourier Transform* (DFT) algorithm in *Raven Pro 1.5 software*^61^. *Discrete Fourier Transform* (DFT) algorithm transforms the same length sequence of equally spaced sample points (N, where N is a prime number) with circular convolution being implemented on the points^62^. All the spectrograms were produced using *Hann windows* at the rate of 1800 samples on 35.2 Hz 3 dB filter (Fig. 2). Only recordings with low levels of background noise and without any overlapping sounds, where the howls were clearly visible as contours, were selected for further analysis. Spectral images were digitised using *Web Plot Digitizer Software*^63^. Thirteen different features (Table 1) were measured from the digitised value by using Microsoft Excel. The details methodology is represented in Fig. 3.

**Table 1:** Thirteen different variables that were measured from the fundamental frequency (f_0_) [Lowest frequency of periodic waveform of each howl].

Variable name	Definition of variable
Min f	The minimum frequency of the fundamental (f_0_)
Max f	The maximum frequency of f_0_
Range f	Range of f_0_; f_0_ = Max f – Min f
Mean f	Mean frequency of f_0_ at 0.1 s interval over the duration
Duration	Duration of Howl measured at f_0_; Duration = t_end_- t_start_
Abrupt_0.025_	Number of abrupt changes in f_0_ more than 25 Hz at single time step (0.1 s)
Abrupt_0.05_	Number of abrupt changes in f_0_ more than 50 Hz at single time step (0.1 s)
Abrupt_0.1_	Number of abrupt changes in f_0_ more than 100 Hz at single time step (0.1 s)
Stdv	Standard deviation of f_0_
Co fm	Coefficient of frequency modulation of f_0_ = Σ|f(t)–f (t + 1)|/(n − 1) × 100/Mean f_0_
Co fv	Coefficient of frequency variation of f_0_ = (SD/mean) × 100
Pos Min	Position in the howl at which the minimum frequency occurs = (time of Minf)/Dur
Pos Max	Position in the howl at which the maximum frequency occurs = (time of Maxf)/Dur

One hundred and thirty-three howls that were longer than 5-s were used for further analysis, with more than ten individual wolves included. The 5 s cut off were chosen to avoid social squeak calls that are very similar to howl but shorter (${\overline{\text{x}}}$ = 3.87 s) and high-frequency variable calls, described by Sadhukhan et al.^55^. Also, the longer howls might contain more identification features than the shorter howls do. *Principal Component Analysis* (PCA) was conducted on measured parameters of 133 howls to reduce the dimension and emphasise the variation between each howl. Out of 133 howls, only 69 howls were identified to an individual. The 69 howls were from nine wolves with known identities: three were captive wolves and six wild, free-ranging wolves, which were identified from their visual features when they were howling in front of the observer and thus howls could be attributed to them individually. The data was further subdivided into training and test datasets. Forty-nine howls from five individuals (2 captives; 3 wild) were used as the training data, and 20 howls from four different individuals (1 captive, 3 wild) as test data to ensure the validity of the method (Table 2). Since the known wolf howls were used test data never used in building model, it provides ‘unbiased sense of model effectiveness’^64^.

**Table 2:** Table showing the information on each individual wolf and their capture date with the number of howls were used in this analysis.

Training/testing	Wolf name	Captive/wild	Capture date	No. of howl
Training data (n = 49)	BMT.SA1	Wild	20/12/2015	5
	CG1.A1	Captive	06/11/2015	3
			08/11/2015	6
	CG2.A1	Captive	05/11/2015	8
			07/11/2015	11
			08/11/2015	9
	GWD.A	Wild	03/02/2016	4
	NNJ.A	Wild	30/01/2016	3
Test data (n = 20)	BMT.A	Wild	19/12/2015	4
	BMT.SA2	Wild	20/12/2015	4
	CG2.A2	Captive	07/11/2015	7
	NU.A	Wild	28/04/2016	5

### Discriminant function analysis

**Figure 4:**
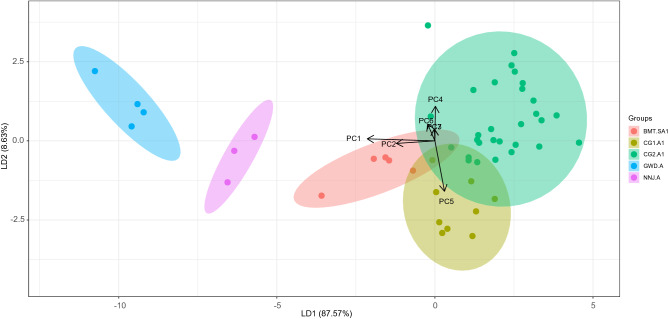
Figure showing a two-dimensional plot of discriminant function analysis using LD1 (Linear Discriminant) and LD2. Each colour represents each wolf. 100% accuracy was achieved in identifying 49 howls from five Indian wolves.

Linear *discriminant function analysis* (DFA) was performed with 49 howls from five individuals (training data) using seven PCA values that contributed more than 5% variation (Table 3) [The cut off value was chosen from scree plot, See Supp. Material 1: PCA.pdf]. The objective of DFA was to construct the linear combination of independent *principal component variables* (PC1–PC7) that will discriminate howls of different individuals. The howls were plotted with discriminant functions at two-dimensional space followed by the group prediction (Fig. 4).

**Table 3:** Table showing the percentage of variation each principal component (PC) accounts for first seven PC function (marked as bold) contributed 94.8% in describing the variable.

	Component importance (%)
**PC1**	**41.2**
**PC2**	**16**
**PC3**	**10.5**
**PC4**	**8.1**
**PC5**	**6.8**
**PC6**	**6.5**
**PC7**	**5.7**
PC8	2
PC9	1.7
PC10	1.1
PC11	0.4
PC12	0
PC13	0

### Hierarchical clustering

To test the success rate of identifying different individuals from their howls with *Linear Discriminant* (LD) score, an *Agglomerative Nesting hierarchical clustering* (AGNES) was executed on 49 howls (training data) that were used in DFA. AGNES initially considers each howl as a different cluster and use a ‘*bottom-up*’ algorithm to join different clusters based on the similarities^65^. The analysis was performed in R using ‘*agnes*’ function in the package *‘dendextend’* and ‘*manhattan*’ metric was used to build the cluster^66^. The same analysis was performed on the test data to determine the accuracy of identifying unknown individuals and estimating the number of wolves from their howls. While the test data contained howls from known individuals, the wolves’ identities were not included in the model. The variables of these 20 howls were calculated from the equation of DFA of 49 howls for cluster analysis.

## Results

### Dimensions reduction to emphasis on variation among howls

**Figure 5:**
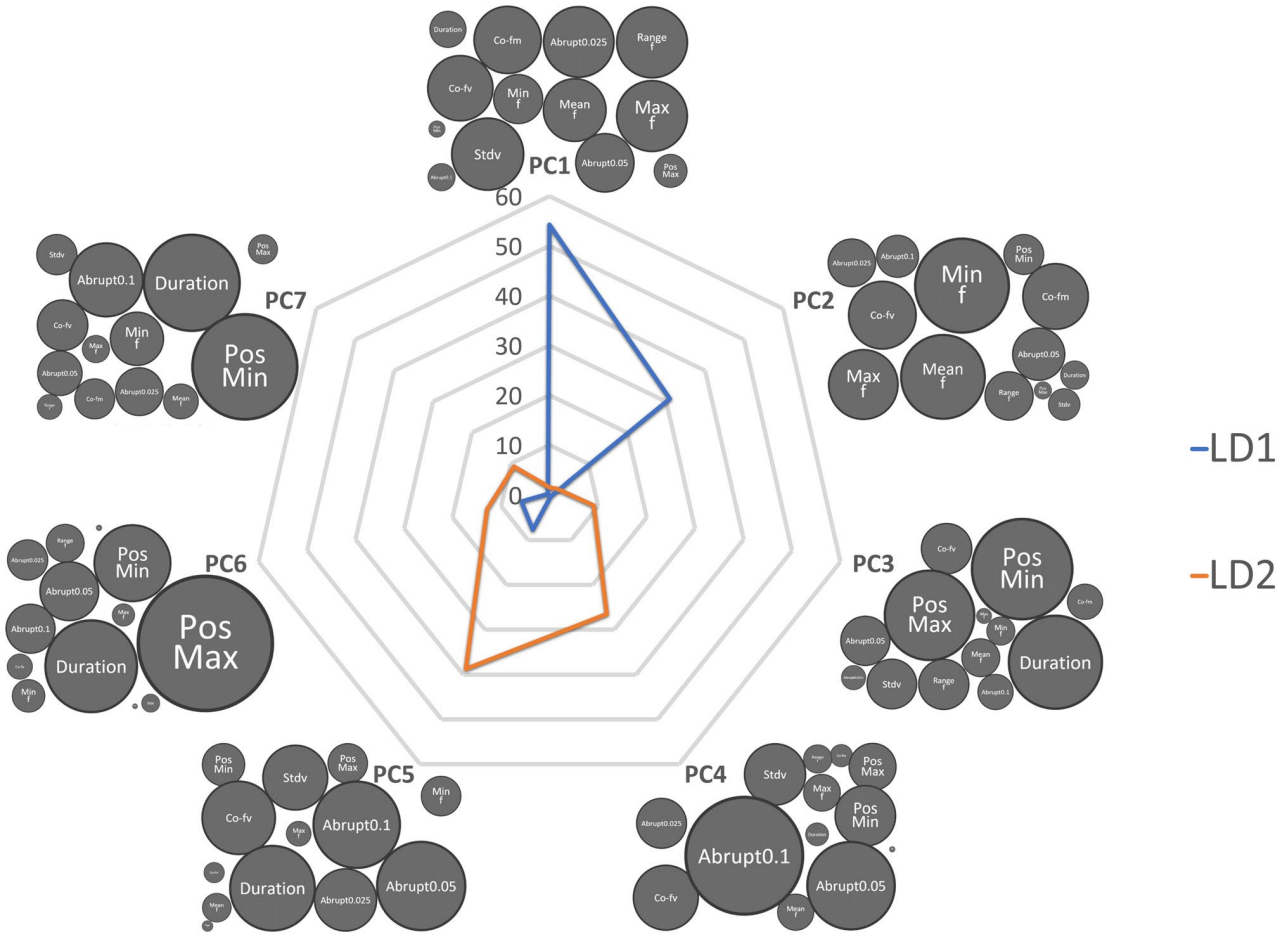
The spider web bubble plot is describing how the *Simple Scalar Variables* (SSV) are ultimately contributing to two LD functions through PC values. The bubble size of each SSV represents the contribution for building each PC function. The blue line represents LD 1, and Orange represents LD2. Since PC1 and PC2 contribute 85% for LD1, the most important SSVs are Stdv f_0_, Min f_0_, Max f_0_ and Mean f_0_. Similarly Duration, Abrupt changes, Co-fv contribute the most in building the LD2 function via PC4 and PC5. LD1 was best defined by the different fundamental frequency factors, while LD2 was best defined through the shape of the frequency contour. Therefore, the critical factors for individuality were encoded in X and Y variables.

Seven *Principal Components* (PC) that explained more than five percent of the variance (Table 3) each were generated from 13 scalar variables (Table 1). These seven PCs together explained 94.8% variance among different howls (Fig. 5). SD of the fundamental frequency (f_0_), Frequency (f_0_) range, Maximum f_0_ and the number of abrupt change (> 25 Hz) were the most important contributing factors for building PC1 which contributed 41.2% explaining the variable (Fig. 5).

### Building discriminant function to emphasis on howl variation among different individuals

The objective of DFA was to build an equation that discriminates the howls of different individuals. The LD score also highlights the variation among howls from different individuals. DFA achieved 100% accuracy in identifying five individuals from 49 howls (Fig. 4). As the first two *Linear Discriminants* (LD1 and LD2) were responsible for 96.2% of the variance to differentiate between howls of different individuals (LD1 = 87.57% and LD2 = 8.63%), we calculated LD1 and LD2 for rest of the howls using the same function (equation) from last DFA. PC1 and PC2 contributed 85% in building LD1; PC4 and PC5 are the most crucial factor (65%) for LD2 function (Fig. 5).

### Identifying individuals from their howls in testing dataset

**Figure 6:**
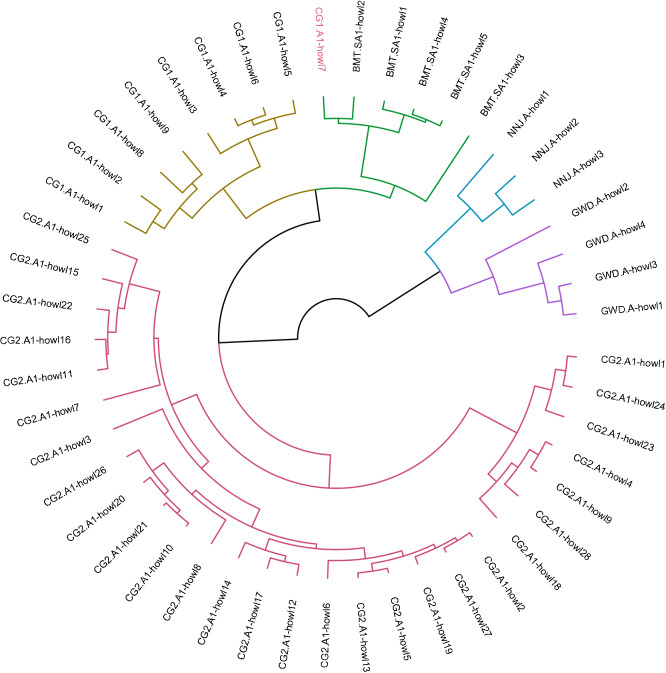
Hierarchical Clustering of 49 howls from five individuals. These 49 howls were used in training the data. 48 howls were identified correctly with the accuracy of 97.9%. The wrongly identified howl is marked in red.

**Figure 7:**
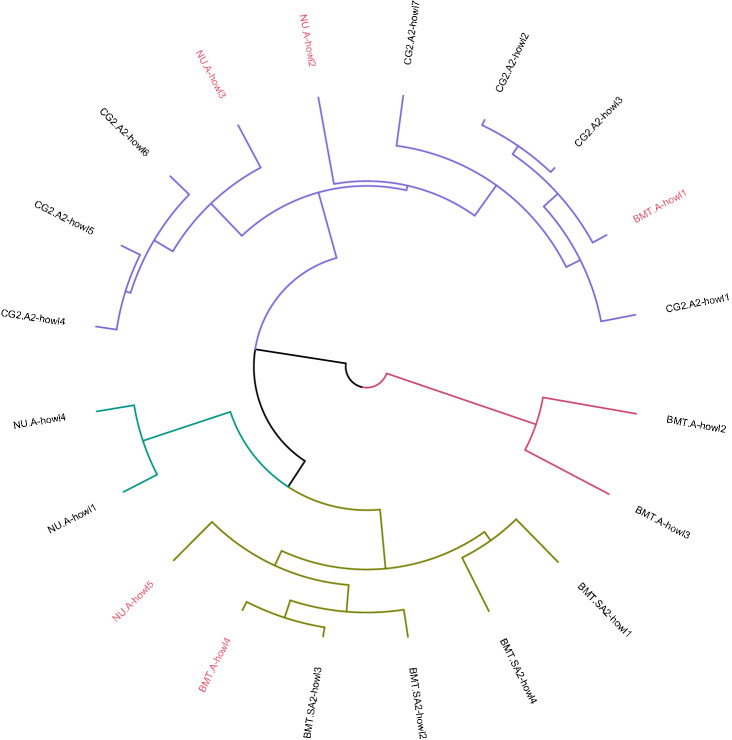
Hierarchical Clustering of 20 howls from four Indian wolves. None of the 20 howls was used in training the data. 15 howls were identified correctly with the accuracy of 75%, and all the four individuals were identified correctly as different clusters. The correctly identified howls are marked in black, and the five wrongly identified howls are marked in red.

First, we tested AGNES on the training dataset (49 howls from 5 individuals) and found 48 howls (~ 97.9% accuracy) were identified correctly at 2.2 clustering scale (Fig. 6). When the same analysis was performed on 20 howls of four different individuals to test the accuracy for the non-training dataset, 15 out of 20 howls from (75% accuracy) four individuals were identified correctly at 2.2 clustering scale (Fig. 7; Table 4). Two howls from wolf BMT.A were misclassified to wolves BMT.SA2 and CG2.A2; Three howls from wolf NU.A were misclassified to wolves BMT.SA2 (1 howl) and CG2.A2 (2 howls) (Fig. 7; Table 4).

**Table 4:** Details of individual identification accuracy using hierarchical clustering on testing data (20 howls from four individual).

Individuals	Predicted group membership				Identification accuracy (correct/total)	Total
	BMT.A	BMT.SA2	CG2.A2	NU.A		
**Count**						
BMT.A	2	1	1	0	2/4	**15/20**
BMT.SA2	0	4	0	0	4/4	
CG2.A2	0	0	7	0	7/7	
NU.A	0	1	2	2	2/5	
**Percent**						
BMT.A	50	25	25	0	50	**75%**
BMT.SA2	0	100	0	0	100	
CG2.A2	0	0	100	0	100	
NU.A	0	20	40	40	40	

15 out of 20 howls were identified correctly with the accuracy of 75%.

## Discussion

Here, we presented a new approach to train the classification model, which can identify individuals from their howls and determine the number of wolves present in a certain number of howls, allowing for fine-scale population surveys. In this study, we built an identification model with known training data which was verified with novel test data. The testing data included howls from the known individuals of both captive and wild Indian wolves but independent from the training dataset so that we can cross-check the identification accuracy without bias. The key finding of our study was 97.9% wolf howls were identified correctly from training data, whereas the accuracy of the model on the testing data was 75%. Moreover, we were able to identify four individuals accurately from the testing dataset. The primary significance of this study is that it can be replicated for any other wolf sub-species with a set of a known wolf howls. This study increases the feasibility of wolf pack census using a howling survey^35,60^. Since wolves may actively avoid camera traps^54^ and photo-identification of wolf requires arduous effort^3,67^, identifying wolves from their howls is a big step towards population estimation using CMR.

Although CMR associated with camera trapping provides population estimation without bias for an identifiable animal like a tiger^68^, camera trapping has several limitations for non-identifiable and long-ranging species like the wolf^3^. Other non-invasive methods like DNA-based CMR resulted in biased population estimation due to the animals’ non-uniform scent-marking patterns^59,69^. However, acoustics based surveys allow vast area sampling with limited resources as compared to camera trapping and other non-invasive methods^3^. Furthermore, our field observations of wolves have shown that the whole pack typically howls during choruses and that all individuals are acoustically active.

For population size estimation through an acoustics-based survey, a combination of CMR and Distance Sampling is required to reduce bias and heterogeneity in detection probability^27,70^. Identifying individual wolves from their howls close this gap of implementing the CMR technique for the population assessment of this elusive and challenging to track species^7,25,27^. While a few studies have established that howls carry individuality information^38^ and known howls can be distinguished from each other^39,45,71^, no study has been successful before in identifying unknown individuals from a set of howls. Furthermore, attempts to count the number of individuals present in a recording have been limited by difficulties in minimising confidence intervals^18,72^. There are two ways to identify individual wolves or packs—supervised clustering and unsupervised clustering. While supervised clustering requires a set of known training data and cluster validation is straightforward, unsupervised clustering requires ground-truthing before it can be used to monitor populations at a survey level and does not allow individual level CMR or tracking^30^.

Although DNA-based identification from faecal sampling is more accurate in identifying individuals than our result, it has drawbacks, such as biased population estimation and the increased cost and effort required to collect and analyse the faeces^59,69^. Nevertheless, the acoustics-based identification model requires further work to increase its accuracy, though we believe that the successful implementation of this method as a CMR-based supervised population estimation model is already possible.

Wolves mostly live in packs that habitually howl together, and it is challenging to identify the specific wolf that is howling, particularly in choruses. If included and incorrectly attributed to a particular wolf, these howls could lead to erroneous predictions by the model. Therefore, this limited our potential data set to those howls which were conclusively attributed to a known individual, and we dropped many howls, especially the chorus howls, from the analysis to avoid misleading the model. However, larger training datasets from different wolf populations might increase the efficacy of the identification model and verification with more wolf howls conceding better reliability as found for Southwestern Willow Flycatcher^73^. Thus, our result of 75% may represent a baseline, not a limit, on the accuracy we could achieve. The inclusion of multiple series of howls from every individual would give a more precise result. However, since none of the free-ranging wolves was radio-collared or marked, this was not possible for the wild wolves. Studying howls of collared wolves would help in adding multiple howl sequences from many free-ranging wolves in the training data and may fill this research gap.

This study revealed that the number of wolves present in the recordings could be determined from their howls and the individuality information is sufficient for supervised population estimation through CMR techniques^7,25,27,30^. Therefore, wolves recorded in one location can be acoustically recaptured at another location, and we can identify them individually. Since our model is exclusively built on fundamental frequency, changes in terrain or vegetation should not affect the accuracy of the model. The information gained from recapturing wolves across different locations would help in deriving territoriality (home-range) information, and this information is crucial for spatially explicit individual-based point process models. This is a clear advancement for developing howling playback surveys as a wolf pack census method. Regular population monitoring will help towards conserving and saving this cryptic species before its population falls beyond a recovery level. Furthermore, since wolf howls can be detected across distances of more than 6 km, identifying wolves from their howls also opens up a new opportunity for non-invasive tracking of this species across large landscapes.

### Guidelines to implement the methodology on the field

We used this methodology to identify individual Indian wolf howl. However, one can use this methodology to identify species, sub-species or individual from their calls. This requires a set of calls to make up the training dataset and a set of calls to make up the testing dataset. We recommend some precautions and step by step guidelines for adapting this method.

I. Before the data collection, one should be cautious about choosing the recorder and data collection methodology. Although we are not definite about the impact of multi-recorder setup in identification accuracy, we recommend using a single microphone set up to keep consistency, especially for individual identification as differences in sensitivity and recording parameters can influence acoustic integrity [See^45^].
II. The multiple groups in the training dataset should be carefully selected to represent distinct group member calls with high confidence (e.g. species/sub-species/individuals), as a single incorrectly identified call in the training dataset can lead the model to erroneous results.
III. The selection of appropriate spectral features is important. While many species encode their identity in the same features, some encoding is species-specific. We tested a wide range of software which fell short in feature extraction for overlapping calls or where background noise was present. The feature description is only as reliable as the extraction. Here, we used *web-plot digitiser* software for spectrogram digitisation. We recommend the use of any semi-automated graph digitiser tool for noisy or overlapping spectral data.
IV. The training data should contain only known groups (multi-species/multiple sub-species/multiple individuals). Each training group should have at least three to five calls and recordings from multiple sessions will increase the accuracy of the model as the animals may have higher intra-individual variation across days than within them. Thus, the higher the intra-individual or intra-group variation, the greater the number of vocalisations and individuals that should be included in the training dataset to make a robust model for the testing dataset.
V. Even though one can choose an unknown dataset as test data, we recommend using a known dataset when originally validating the model. Using multiple test datasets will increase the model’s confidence.
VI. We recommend using multiple small batches as test data (50–100 sample of calls) instead of large data to avoid confusion in cluster groups that may represent other variation in the calls.
VII. To allow study replication, we have made our data and codes available in the Supplementary Materials. While the data needs to be replaced for each study, the system of analysis and classification should be robust and replicable.

## Conclusion

Our study reached substantial accuracy in identifying wolves from their howls. Since the methodology was validated using known wolf data and was found to be reasonably reliable, unknown howls can also be classified. This opens up a new opportunity for population estimation and tracking of wolves through howling surveys. Although we analysed our data with Indian wolf howls, the procedure is replicable for other subspecies that have a set of known howls from different individuals and could potentially be applied to other species with individually distinctive vocalisations. This would refine and improve both population estimates and the ability to monitor individuals in situ, with global implications for conservation and ecology.

## Appendices: Supplementary materials

All the data and R code require to recreate the analysis are hosted in https://github.com/bhlabwii/wolf_howlID platform. Raw sound files are available on request to the corresponding author. Compiled reports from R Scripts can be found in following supporting material:

**Table:** 

Filename	Description
PCA.pdf	Principal Component Analysis of 133 howl
DFA.49H5ID.PCvalue.pdf	Discriminant Function Analysis of 49 howls from five individuals
known_dend_49H5ID.pdf	Agglomerative Nesting hierarchical clustering (AGNES) using 49 howls from five individuals
Dendrogram.test.pdf	Agglomerative Nesting hierarchical clustering (AGNES) using 20 howls from four different individuals to test the model

### Supplementary Information

Supplementary Information 1.

Supplementary Information 2.

Supplementary Information 3.

Supplementary Information 4.
