# Supplementary material for: Identifying unknown Indian wolves by their distinctive howls: its potential as a non-invasive survey method
Source: Sci Rep. 2021 Mar 31;11:7309. doi: 10.1038/s41598-021-86718-w (PMC8012383; doi:10.1038/s41598-021-86718-w)
Supplement: Supplementary file 1 — Supplementary Information 1. [file 41598_2021_86718_MOESM1_ESM.pdf]

Supplemental Materials for

Identifying unknown Indian wolves by their distinctive howls: its potential as a non-invasive survey method

Sougata Sadhukhan [a], Holly Root-Gutteridge[b, c], and Bilal Habib [a]*

[a]*Animal Ecology and Conservation Biology, Wildlife Institute of India, Dehradun, India;* [b]*Animal Behaviour, Cognition and Welfare Group, University of Lincoln, Lincoln, UK;* [c]*Reby Lab, School of Psychology, University of Sussex, Brighton, UK*

* Corresponding Author

Scientists-E, Wildlife Institute of India, Dehradun-248001, India, Email id- bh@wii.gov.in

PCA Analysis

(PCA.pdf)

Principal Component Analysis of 133 howl

```
#set directory
setwd
("D:/Wolf_Project/Howl_recognise_fresh20200821_1133/Analysis/R/PCA.133H")
library(readxl)

#readfile
howl133 <- read_excel("133H.xlsx")
str(howl133)

## tibble [133 x 16] (S3: tbl_df/tbl/data.frame)
##  $ Filename            : chr [1:133] "151219-001_Baramati1_A1" "151219-
001_Baramati1_A2" "151219-001_BaramatiX_A4" "151219-001_BaramatiX_A5" ...
##  $ ID                  : chr [1:133] "BMT.A" "BMT.A" "BMT.A" "BMT.A" ...
##  $ Series name         : chr [1:133] "A1" "A2" "A4" "A5" ...
##  $ MeanF               : num [1:133] 0.34 0.421 0.447 0.423 0.411 ...
##  $ Max of F            : num [1:133] 0.365 0.549 0.579 0.53 0.468 ...
##  $ Min of F            : num [1:133] 0.304 0.301 0.295 0.31 0.349 ...
##  $ FRange              : num [1:133] 0.0255 0.128 0.1323 0.1069 0.0571
...
```

```
## $ StdDevF              : num [1:133] 0.0138 0.0679 0.093 0.0545 0.0265
...
## $ Duration(T)          : num [1:133] 6.63 7.18 6.44 6.04 11.25 ...
## $ Sum of Abrupt(>0.025): num [1:133] 0 4 9 3 2 0 0 0 0 2 ...
## $ Sum of Abrupt(>0.05) : num [1:133] 0 0 0 0 0 0 0 0 0 0 ...
## $ Sum of Abrupt(>0.1)  : num [1:133] 0 0 0 0 0 0 0 0 0 0 ...
## $ Cofm                 : num [1:133] 0.873 1.54 2.243 1.708 1.626 ...
## $ CofFV                : num [1:133] 4.06 16.12 20.8 12.89 6.44 ...
## $ PosMax               : num [1:133] 0.633 0.534 0.482 0.612 0.405 ...
## $ PosMin               : num [1:133] 0.0355 0 0 0.8274 0 ...
```

```
howl133_variable <- howl133 [,4:16]
howl133_variable
```

```
## Warning: `...` is not empty.
##
## We detected these problematic arguments:
## * `needs_dots`
##
## These dots only exist to allow future extensions and should be empty.
## Did you misspecify an argument?
```

```
## # A tibble: 133 x 13
##    MeanF `Max of F` `Min of F` FRange StdDevF `Duration(T)` `Sum of
Abrupt(~
##    <dbl>      <dbl>      <dbl>  <dbl>   <dbl>         <dbl>
<dbl>
##  1 0.340      0.365      0.304 0.0255  0.0138          6.63
0
##  2 0.421      0.549      0.301 0.128   0.0679          7.18
4
##  3 0.447      0.579      0.295 0.132   0.0930          6.44
9
##  4 0.423      0.530      0.310 0.107   0.0545          6.04
3
##  5 0.411      0.468      0.349 0.0571  0.0265         11.3
2
##  6 0.415      0.462      0.356 0.0467  0.0239         11.2
0
##  7 0.453      0.517      0.345 0.0648  0.0376          7.97
0
##  8 0.400      0.444      0.324 0.0439  0.0285          5.12
0
##  9 0.400      0.443      0.328 0.0429  0.0278          5.10
0
## 10 0.406      0.533      0.335 0.128   0.0490          9.87
2
## # ... with 123 more rows, and 6 more variables: `Sum of Abrupt(>0.05)`
<dbl>,
```

```
## #   `Sum of Abrupt(>0.1)` <dbl>, Cofm <dbl>, CofFV <dbl>, PosMax <dbl>,
## #   PosMin <dbl>

pca1 <- prcomp(howl133_variable, scale=TRUE)

#ploting PCA pca1$x[,1] for component 1 & pca1$x [,2] for comp 2
plot(pca1$x[,1], pca1$x [,2])
```

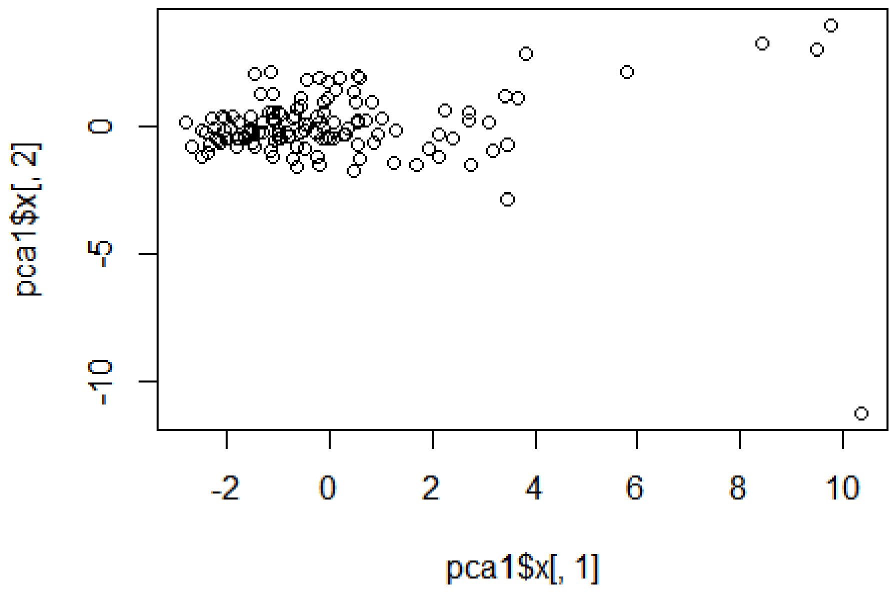


```
#exporting PC values
PC1 <-pca1$x[,1]
PC2 <-pca1$x[,2]
PC3 <-pca1$x[,3]
PC4 <-pca1$x[,4]
PC5 <-pca1$x[,5]
PC6 <-pca1$x[,6]
PC7 <-pca1$x[,3]

PC_score <- data.frame(howl133$Filename, PC1, PC2, PC3, PC4, PC5, PC6, PC7)
write.csv(PC_score, "PCA.score.133H.csv")

pca1.var <-pca1$sdev^2
pca1.var.per <-round(pca1.var/sum(pca1.var)*100,1)
write.csv(pca1.var.per, "PCA_components_imp.csv")

barplot(pca1.var.per, main="Scree Plot", xlab="Principal Component", ylab =
"percent variation", names.arg=TRUE)
```

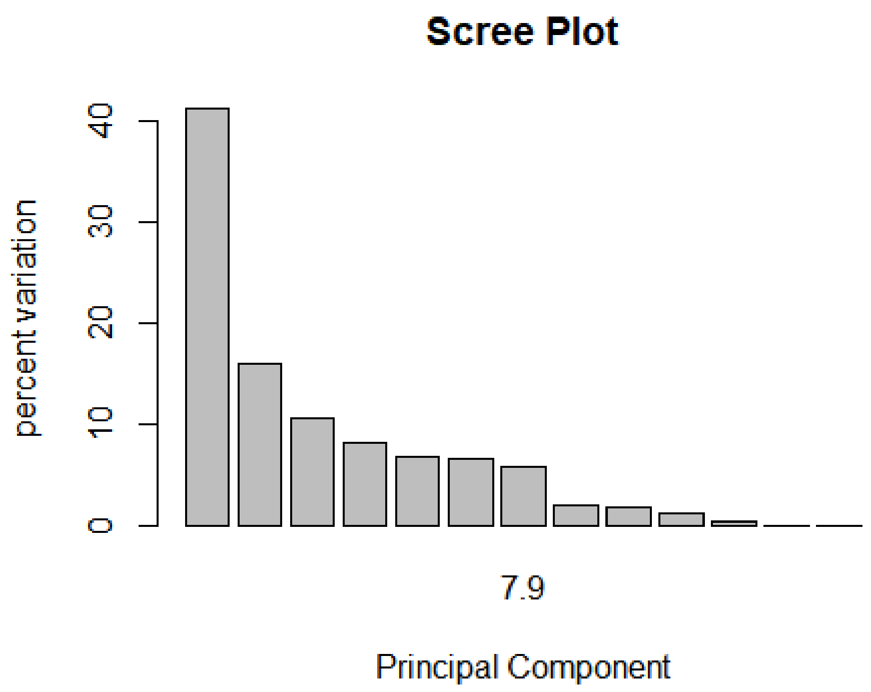


```
#loading_scores for PC1
loading_scores <-pca1$rotation[,2]
variable_score <- abs(loading_scores)
variable_score_ranked <- sort(variable_score, decreasing = TRUE)
variable_score_ranked

##            Min of F                MeanF              Max of F
##          0.60146647           0.48011579            0.32741802
##               CofFV                 Cofm  Sum of Abrupt(>0.05)
##          0.31043518           0.29265351            0.18593125
##              FRange Sum of Abrupt(>0.025)   Sum of Abrupt(>0.1)
##          0.15849518           0.15528872            0.12081324
##              PosMin              StdDevF           Duration(T)
##          0.10958284           0.06823755            0.05493410
##              PosMax
##          0.02059558

#export PC-rotation value
colnames(howl133_variable)

##  [1] "MeanF"                 "Max of F"              "Min of F"
##  [4] "FRange"                "StdDevF"               "Duration(T)"
##  [7] "Sum of Abrupt(>0.025)" "Sum of Abrupt(>0.05)"  "Sum of Abrupt(>0.1)"
## [10] "Cofm"                  "CofFV"                 "PosMax"
## [13] "PosMin"
```

```r
PC_load<-
data.frame(colnames(howl133_variable),pca1$rotation[,1],pca1$rotation[,2],pca
1$rotation[,3],

pca1$rotation[,4],pca1$rotation[,5],pca1$rotation[,6],pca1$rotation[,7])
write.csv(PC_load, "variable_score_PCA.csv")

#loading_scores for PC2
loading_scores_pc2 <-pca1$rotation[,2]
variable_score_pc2 <- abs(loading_scores_pc2)
variable_score_pc2_ranked <- sort(variable_score_pc2, decreasing = TRUE)
variable_score_pc2_ranked

##              Min of F                  MeanF                Max of F
##            0.60146647             0.48011579              0.32741802
##                 CofFV                   Cofm  Sum of Abrupt(>0.05)
##            0.31043518             0.29265351              0.18593125
##                FRange Sum of Abrupt(>0.025)   Sum of Abrupt(>0.1)
##            0.15849518             0.15528872              0.12081324
##                PosMin                StdDevF             Duration(T)
##            0.10958284             0.06823755              0.05493410
##                PosMax
##            0.02059558
```
