# Supplementary material for: Identifying unknown Indian wolves by their distinctive howls: its potential as a non-invasive survey method
Source: Sci Rep. 2021 Mar 31;11:7309. doi: 10.1038/s41598-021-86718-w (PMC8012383; doi:10.1038/s41598-021-86718-w)
Supplement: Supplementary file 2 — Supplementary Information 2. [file 41598_2021_86718_MOESM2_ESM.pdf]

Supplemental Materials for

Identifying unknown Indian wolves by their distinctive howls: its potential as a non-invasive survey method

Sougata Sadhukhan [a], Holly Root-Gutteridge[b, c], and Bilal Habib [a]*

[a]*Animal Ecology and Conservation Biology, Wildlife Institute of India, Dehradun, India;* [b]*Animal Behaviour, Cognition and Welfare Group, University of Lincoln, Lincoln, UK;* [c]*Reby Lab, School of Psychology, University of Sussex, Brighton, UK*

* Corresponding Author

Scientists-E, Wildlife Institute of India, Dehradun-248001, India, Email id- bh@wii.gov.in

DFA Analysis

(DFA.49H5ID.PCvalue.pdf)

Discriminant Function Analysis of 49 howls from five individuals

```
#install.packages("psych")

# load package
library(readxl)
library(psych)

#working directory from Options | General
setwd
("D:/Wolf_Project/Howl_recognise_fresh20200821_1133/Analysis/R/DFA_50H")

#Reading excel file
howl_3ID <- read_excel("50H5ID.xlsx")
#howl_3ID

#Assign Factors
howl_3ID$Individual=as.factor(howl_3ID$Individual)
#levels(howl_3ID$Individual)
#str(howl_3ID)
```

```
#LDA
library(MASS)
LDAhowl <- lda(formula = Individual ~ PC1+PC2+PC3+PC4+PC5+PC6+PC7,
               data=howl_3ID)

## Warning in lda.default(x, grouping, ...): variables are collinear

LDAhowl

## Call:
## lda(Individual ~ PC1 + PC2 + PC3 + PC4 + PC5 + PC6 + PC7, data = howl_3ID)
##
## Prior probabilities of groups:
## BMT.SA1  CG1.A1  CG2.A1   GWD.A   NNJ.A
##    0.10    0.18    0.58    0.08    0.06
##
## Group means:
##                 PC1        PC2         PC3         PC4         PC5
PC6
## BMT.SA1  0.06392947  0.4973106  0.18247862 -0.04631654  0.56476492 -
0.06886681
## CG1.A1  -0.13804595 -1.2008510 -1.11871278 -0.37933573  0.57659790 -
0.03434849
## CG2.A1  -1.49687697 -0.2846193  0.19141176  0.02661182 -0.19289132
0.51008743
## GWD.A    3.21610497  1.5176549 -0.72903321  2.55021011  0.73863535
0.05368546
## NNJ.A    1.61255614  1.2525660  0.06990513 -0.44711814 -0.08666472 -
0.40393601
##                 PC7
## BMT.SA1  0.18247862
## CG1.A1  -1.11871278
## CG2.A1   0.19141176
## GWD.A   -0.72903321
## NNJ.A    0.06990513
##
## Coefficients of linear discriminants:
##             LD1         LD2        LD3         LD4
## PC1 -2.14077851  0.06420895 -0.3140400  0.35732770
## PC2 -1.21923185 -0.08183570  1.2565775 -0.33729595
## PC3 -0.01774952  0.37593437  0.1022556  0.01782616
## PC4  0.01500758  1.09224773 -1.0354120 -0.10368951
## PC5  0.30345377 -1.59455937  0.8421461 -0.96016925
## PC6 -0.23008852  0.53052336 -0.1070340  0.03593903
## PC7 -0.01774952  0.37593437  0.1022556  0.01782616
##
## Proportion of trace:
##    LD1    LD2    LD3    LD4
## 0.8757 0.0863 0.0355 0.0025
```

```
#histogram
p <- predict(LDAhowl, howl_3ID)


px<-data.frame(howl_3ID$Filename, howl_3ID$Individual, p)
write.csv(px, "howlpredict_train.result.csv")
write

## function (x, file = "data", ncolumns = if (is.character(x)) 1 else 5,
##     append = FALSE, sep = " ")
## cat(x, file = file, sep = c(rep.int(sep, ncolumns - 1), "\n"),
##     append = append)
## <bytecode: 0x000000000e3d6d20>
## <environment: namespace:base>

par(mar=c(1,1,1,1))
ldahist (data =p$x[,1:1], g = howl_3ID$Individual)
```

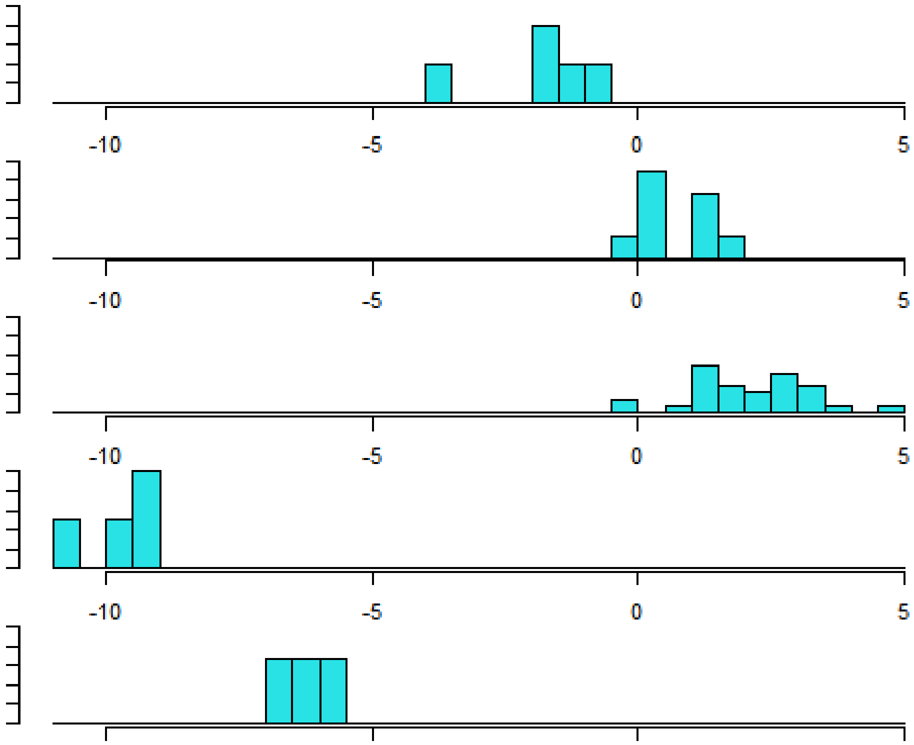


```
ldahist (data =p$x[,2:2], g = howl_3ID$Individual)
```

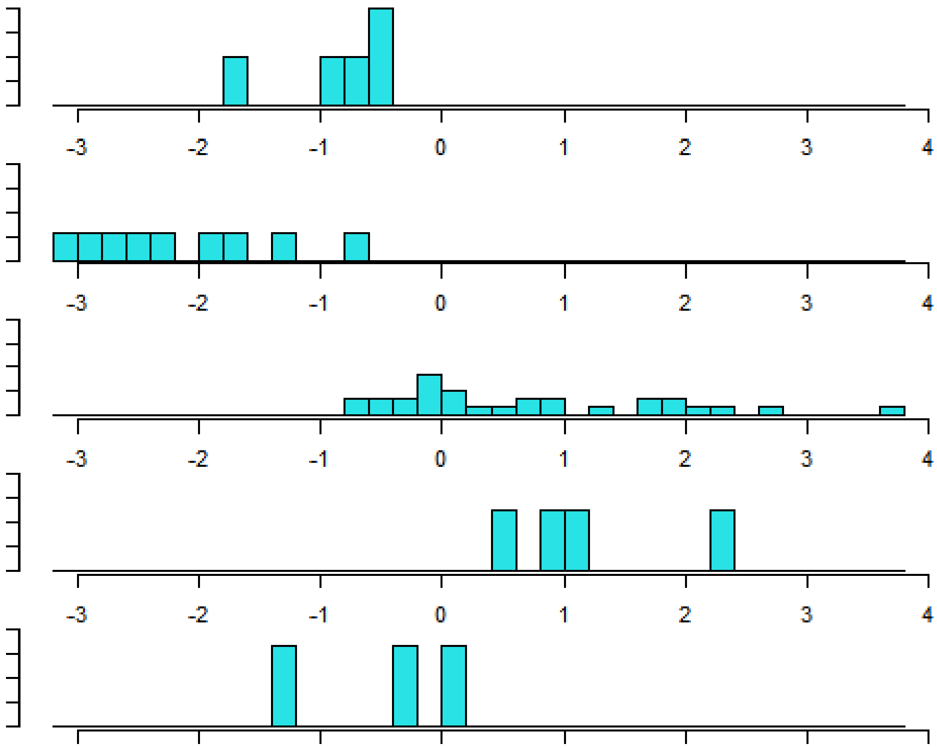


```
#Bi-plot
#install.packages("devtools")
#install.packages("ps", dependencies = T)
#install_github("fawda123/ggord")

library(devtools)

## Loading required package: usethis

library(ggord)
ggord(LDAhowl, howl_3ID$Individual,
      xlim = c(-12, 5),
      ylim = c (-4.5, 4))
```

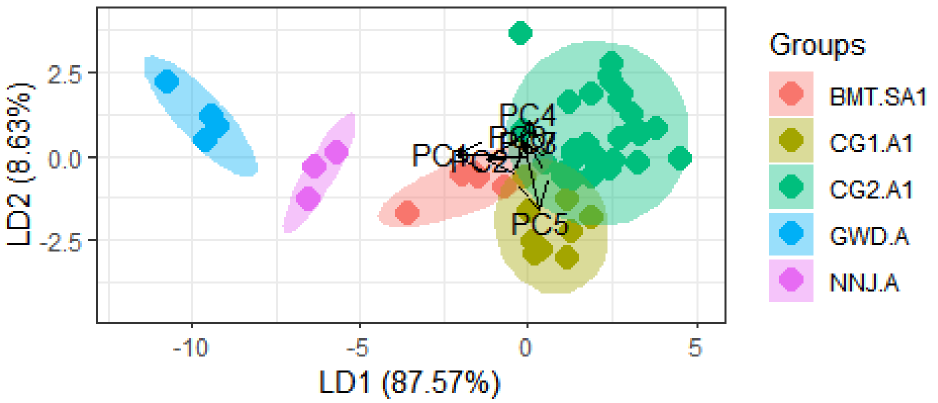


```
# Confusion matrix and accuracy - training data
p1 <- predict(LDAhowl, howl_3ID)$class
tab <- table(Predicted = p1, Actual = howl_3ID$Individual)
tab

##           Actual
## Predicted BMT.SA1 CG1.A1 CG2.A1 GWD.A NNJ.A
##   BMT.SA1       5      0      0     0     0
##   CG1.A1        0      9      0     0     0
##   CG2.A1        0      0     29     0     0
##   GWD.A         0      0      0     4     0
##   NNJ.A         0      0      0     0     3

sum(diag(tab))/sum(tab)

## [1] 1

# Confusion matrix and accuracy - testing data
howl_test <- read_excel("Testing.xlsx")

#calculate value LD value for all howl
p3 <- predict(LDAhowl, howl_test)
p3x<-data.frame(howl_test$Filename, howl_test$Individual, p3)
write.csv(p3x, "howlpredict_test_LDscore .results.csv")
```
