# Supplementary material for: Identifying unknown Indian wolves by their distinctive howls: its potential as a non-invasive survey method
Source: Sci Rep. 2021 Mar 31;11:7309. doi: 10.1038/s41598-021-86718-w (PMC8012383; doi:10.1038/s41598-021-86718-w)

Supplemental Materials for

Identifying unknown Indian wolves by their distinctive howls: its potential as a non-invasive survey method

Sougata Sadhukhan [a], Holly Root-Gutteridge[b, c], and Bilal Habib [a]*

[a]*Animal Ecology and Conservation Biology, Wildlife Institute of India, Dehradun, India;* [b]*Animal Behaviour, Cognition and Welfare Group, University of Lincoln, Lincoln, UK;* [c]*Reby Lab, School of Psychology, University of Sussex, Brighton, UK*

* Corresponding Author

Scientists-E, Wildlife Institute of India, Dehradun-248001, India, Email id- bh@wii.gov.in

Dendrogram Analysis (Train Data)

(known_dend_49H5ID.pdf)

Agglomerative Nesting hierarchical clustering (AGNES) using 49 howls from five individuals

```
setwd
("D:/Wolf_Project/Howl_recognise_fresh20200821_1133/Analysis/R/Dendogram_know
n")

#Required Packages
#install.packages("dendextend")
#install.packages("circlize")

# load package cluster
library(cluster, quietly = TRUE)
library(readxl)
library(dendextend)

##
## ---------------------
## Welcome to dendextend version 1.14.0
## Type citation('dendextend') for how to cite the package.
##
```

```
## Type browseVignettes(package = 'dendextend') for the package vignette.
## The github page is: https://github.com/talgalili/dendextend/
##
## Suggestions and bug-reports can be submitted at:
https://github.com/talgalili/dendextend/issues
## Or contact: <tal.galili@gmail.com>
##
##  To suppress this message use:
suppressPackageStartupMessages(library(dendextend))
## ---------------------

##
## Attaching package: 'dendextend'

## The following object is masked from 'package:stats':
##
##     cutree

library(colorspace) # get nice colors
library(circlize)

## ========================================
## circlize version 0.4.10
## CRAN page: https://cran.r-project.org/package=circlize
## Github page: https://github.com/jokergoo/circlize
## Documentation: https://jokergoo.github.io/circlize_book/book/
##
## If you use it in published research, please cite:
## Gu, Z. circlize implements and enhances circular visualization
##   in R. Bioinformatics 2014.
##
## This message can be suppressed by:
##   suppressPackageStartupMessages(library(circlize))
## ========================================

#Reading excel file
wolf49H <- read_excel("49H5ID_LDScroe.xlsx")

## New names:
## * `` -> ...1

str(wolf49H)

## tibble [49 x 13] (S3: tbl_df/tbl/data.frame)
##  $ ...1                : num [1:49] 1 2 3 4 5 6 7 8 9 10 ...
##  $ howl_3ID.Filename   : chr [1:49] "160203-001_Gangewadi2_A2(GWD.A)"
"160203-001_Gangewadi2_A3(GWD.A)" "160203-001_Gangewadi2_A4(GWD.A)" "160203-
001_Gangewadi2_A5(GWD.A)" ...
##  $ howl_3ID.Individual : chr [1:49] "GWD.A" "GWD.A" "GWD.A" "GWD.A" ...
##  $ class               : chr [1:49] "Gangewadi2" "Gangewadi2" "Gangewadi2"
"Gangewadi2" ...
##  $ posterior.Baramati18: num [1:49] 1.57e-13 1.84e-22 9.75e-15 6.36e-15
```

```
1.64e-06 ...
##  $ posterior.CG1       : num [1:49] 2.70e-23 3.03e-33 1.32e-24 1.80e-24
4.74e-06 ...
##  $ posterior.CG2       : num [1:49] 3.14e-27 1.88e-37 1.95e-28 2.56e-29
1.00 ...
##  $ posterior.Gangewadi2: num [1:49] 9.97e-01 1.00 1.00 1.00 4.43e-35 ...
##  $ posterior.Nanaj1    : num [1:49] 2.73e-03 2.45e-12 3.89e-04 4.41e-04
6.86e-19 ...
##  $ x.LD1               : num [1:49] -9.21 -10.75 -9.41 -9.59 2.53 ...
##  $ x.LD2               : num [1:49] 0.903 2.203 1.161 0.457 2.188 ...
##  $ x.LD3               : num [1:49] -0.195 -3.401 -0.487 -0.646 1.658 ...
##  $ x.LD4               : num [1:49] 1.561 -5.157 1.545 1.611 -0.586 ...
```

```
wolf49Hx <- wolf49H [,10:13]
wolf49Hx
```

```
## Warning: `...` is not empty.
##
## We detected these problematic arguments:
## * `needs_dots`
##
## These dots only exist to allow future extensions and should be empty.
## Did you misspecify an argument?
```

```
## # A tibble: 49 x 4
##     x.LD1   x.LD2  x.LD3   x.LD4
##     <dbl>   <dbl>  <dbl>   <dbl>
##  1  -9.21  0.903  -0.195  1.56
##  2 -10.8   2.20   -3.40  -5.16
##  3  -9.41  1.16   -0.487  1.54
##  4  -9.59  0.457  -0.646  1.61
##  5   2.53  2.19    1.66  -0.586
##  6   1.40 -0.0601 -0.877  0.615
##  7   4.56 -0.0561 -2.10   0.0430
##  8   2.78  1.82   -0.178  0.363
##  9   1.85  0.0228  0.476 -0.370
## 10   1.78  0.372   0.523 -0.233
## # ... with 39 more rows
```

```
wolf49H.rn <- data.frame (wolf49H$howl_3ID.Filename, wolf49Hx$x.LD1,
wolf49Hx$x.LD2, row.names = TRUE )
wolf49H.rn
```

```
##                                     wolf49Hx.x.LD1 wolf49Hx.x.LD2
## 160203-001_Gangewadi2_A2(GWD.A)      -9.21156837     0.90313345
## 160203-001_Gangewadi2_A3(GWD.A)     -10.75337408     2.20315993
## 160203-001_Gangewadi2_A4(GWD.A)      -9.40668175     1.16131755
## 160203-001_Gangewadi2_A5(GWD.A)      -9.59212817     0.45745107
## 151108-000_CG2_A1.(CG2.A1)            2.52627042     2.18766673
## 151108-000_CG2_A10.(CG2.A1)           1.39984169    -0.06010067
## 151108-000_CG2_A11.(CG2.A1)           4.55625388    -0.05611113
```

```
## 151108-000_CG2_A2(CG2.A1)             2.77681825     1.82411998
## 151108-000_CG2_A3.(CG2.A1)            1.85177408     0.02279588
## 151108-000_CG2_A5.(CG2.A1)            1.77810416     0.37209610
## 151108-000_CG2_A7.(CG2.A1)           -0.13157598     0.77132734
## 151108-000_CG2_A8.(CG2.A1)            0.51519971    -0.20805478
## 151108-000_CG2_A9.(CG2.A1)            2.76399008     1.64391012
## 151106-002_CG1_A1(CG1.A1)             1.89234100    -1.83479237
## 151106-002_CG1_A2(CG1.A1)             1.30086239    -2.22850576
## 151106-002_CG1_A3(CG1.A1)             1.18664870    -3.00721264
## 151108-001_CG1_A2.(CG1.A1)            0.23157374    -2.91075681
## 151108-001_CG1_A3(CG1.A1)             0.13733401    -2.57100907
## 151108-001_CG1_A3.(CG1.A1)            0.39510610    -2.77776500
## 151108-001_CG1_A4.(CG1.A1)           -0.07785398    -0.60765603
## 151108-001_CG1_B3.(CG1.A1)            0.03955782    -1.61934952
## 151108-001_CG1_B4.(CG1.A1)            1.14940919    -1.27755514
## 151105-001_CG2_1(CG2.A1)              1.06602207    -0.61675755
## 151105-001_CG2_11.(CG2.A1)            3.20415489     0.85429171
## 151105-001_CG2_12.(CG2.A1)            2.55287252    -0.12365858
## 151105-001_CG2_2.(CG2.A1)             2.04725063    -0.01788157
## 151105-001_CG2_3(CG2.A1)              3.30049628    -0.18056351
## 151105-001_CG2_5.(CG2.A1)             2.72542120     0.52779559
## 151105-001_CG2_6.(CG2.A1)             3.37210027     0.64962085
## 151105-001_CG2_7.(CG2.A1)             2.42376081    -0.35624135
## 151107-001_CG2_A1(CG2.A1)             1.21709235     1.60669355
## 151107-001_CG2_A10(CG2.A1)            1.37050897     0.18081623
## 151107-001_CG2_A11.(CG2.A1)           1.33897832    -0.67366753
## 151107-001_CG2_A12(CG2.A1)            1.06662916    -0.52185213
## 151107-001_CG2_A2(CG2.A1)             3.84789416     0.80380468
## 151107-001_CG2_A3(CG2.A1)             2.50915277     2.77291605
## 151107-001_CG2_A4(CG2.A1)             2.42372938     2.38570007
## 151107-001_CG2_A5.(CG2.A1)            3.11728650     1.27185615
## 151107-001_CG2_A6.(CG2.A1)            1.91299548    -0.59700402
## 151107-001_CG2_A7.(CG2.A1)            1.33691033     0.03027727
## 151107-001_CG2_A9.(CG2.A1)            1.89305945     1.84944259
## 151220-002_Baramati18_A1(BMT.SA1)    -1.93311680    -0.56704888
## 151220-002_Baramati18_A2(BMT.SA1)    -0.69250563    -0.94015587
## 151220-002_Baramati18_A3(BMT.SA1)    -3.58516962    -1.73174889
## 151220-002_Baramati18_B1(BMT.SA1)    -1.44887816    -0.61828805
## 151220-002_Baramati18_C1(BMT.SA1)    -1.56373678    -0.52333323
## 160130-001_Nanaj1_A1(NNJ.A)          -6.55877100    -1.31021107
## 160130-001_Nanaj1_A2(NNJ.A)          -6.34602012    -0.31523229
## 160130-001_Nanaj1_A3(NNJ.A)          -5.70535335     0.12568310
```

```
str(wolf49H.rn)
```

```
## 'data.frame':    49 obs. of  2 variables:
##  $ wolf49Hx.x.LD1: num  -9.21 -10.75 -9.41 -9.59 2.53 ...
##  $ wolf49Hx.x.LD2: num  0.903 2.203 1.161 0.457 2.188 ...
```

```r
agn2 <- agnes(wolf49H.rn, metric = "manhattan", stand = TRUE)
dend <- as.dendrogram(agn2)

dend <- as.dendrogram(agn2)

# order it the closest we can to the order of the observations:
dend <- rotate(dend, 1:0)

## Warning in weights_for_order[order_x[order]] <- weights: number of items
to
## replace is not a multiple of replacement length

# Color the branches based on the hight:
dend <- color_branches(dend, h= 2.2) #, groupLabels=different Howling
Individual)

# reduce the size of the labels:
# dend <- assign_values_to_leaves_nodePar(dend, 0.5, "lab.cex")
dend <- set(dend, "labels_cex", .5)
# And plot:
par(mar=c(1,1,1,1),  mgp=c(1, 1, 1))
circlize_dendrogram(dend, lebels=TRUE, row.names= TRUE, labels_track_height =
0.4)
```

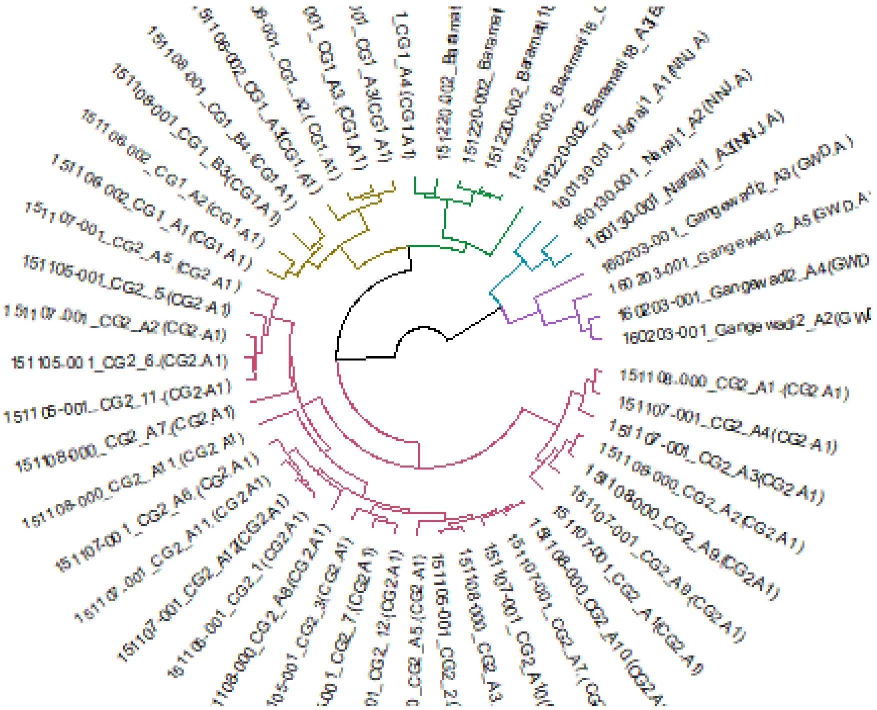

```r
dend

## 'dendrogram' with 2 branches and 49 members total, at height 4.904895
```

```
#writetable
clustnumber<- cutree(dend, k=5)
dendogram_50known_howl <-data.frame(wolf49H$howl_3ID.Individual, clustnumber)
write.csv(dendogram_50known_howl, "49h5ID_dend_results.csv")

#########################
agn3 <- agnes(wolf49H.rn, metric = "manhattan", stand = TRUE)

dend2 <- as.dendrogram(agn3)
# order it the closest we can to the order of the observations:
dend2 <- rotate(dend2, 1:0)

## Warning in weights_for_order[order_x[order]] <- weights: number of items
to
## replace is not a multiple of replacement length

# Color the branches based on the hight:
dend2 <- color_branches(dend2, h= 2.2) #, groupLabels=different Howling
Individual)

# reduce the size of the labels:
# dend <- assign_values_to_leaves_nodePar(dend, 0.5, "lab.cex")
dend2 <- set(dend2, "labels_cex", .5)
#colour highlighted row
highlight<- row.names(wolf49H.rn)[20]
highlight

## [1] "151108-001_CG1_A4.(CG1.A1)"

dend2 <- color_labels(dend2, labels = highlight , col = 2)
# And plot:
par(mar=c(1,1,1,1),  mgp=c(1, 1, 1))

circlize_dendrogram(dend2, lebels=TRUE, row.names= TRUE, labels_track_height
= 0.4)
```

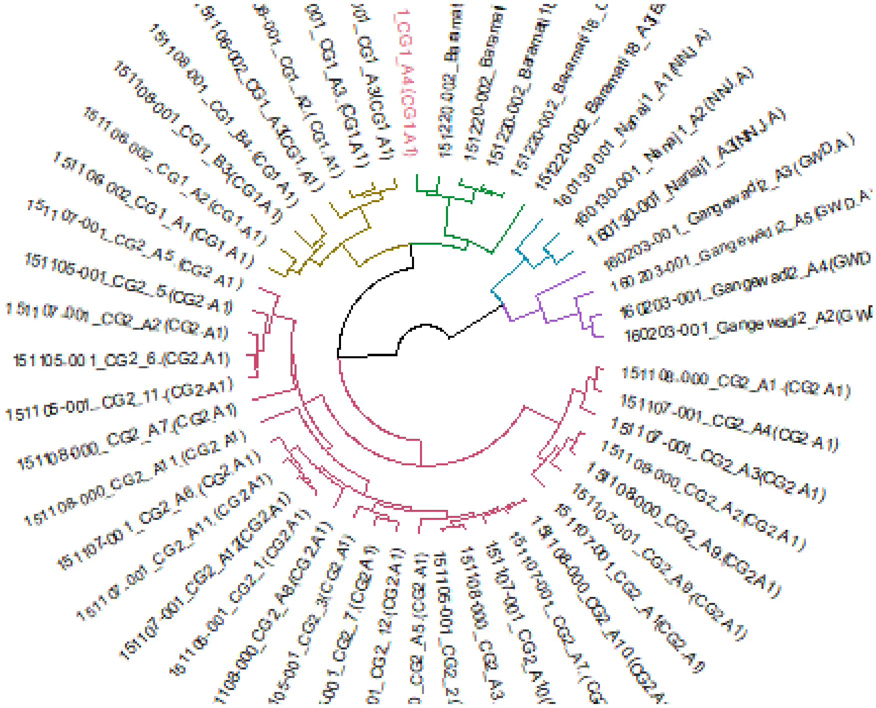

Supplement: Supplementary file 3 — Supplementary Information 3. [file 41598_2021_86718_MOESM3_ESM.pdf]
