# Supplementary material for: Identifying unknown Indian wolves by their distinctive howls: its potential as a non-invasive survey method
Source: Sci Rep. 2021 Mar 31;11:7309. doi: 10.1038/s41598-021-86718-w (PMC8012383; doi:10.1038/s41598-021-86718-w)

Supplemental Materials for

Identifying unknown Indian wolves by their distinctive howls: its potential as a non-invasive survey method

Sougata Sadhukhan [a], Holly Root-Gutteridge[b, c], and Bilal Habib [a]*

[a]*Animal Ecology and Conservation Biology, Wildlife Institute of India, Dehradun, India;* [b]*Animal Behaviour, Cognition and Welfare Group, University of Lincoln, Lincoln, UK;* [c]*Reby Lab, School of Psychology, University of Sussex, Brighton, UK*

* Corresponding Author

Scientists-E, Wildlife Institute of India, Dehradun-248001, India, Email id- bh@wii.gov.in

Dendrogram Analysis (Test Data)

(Dendrogram.test.pdf)

Agglomerative Nesting hierarchical clustering (AGNES) using 20 howls from four different individuals to test the model

```
setwd
("D:/Wolf_Project/Howl_recognise_fresh20200821_1133/Analysis/R/Dendogram_test
")

#Required Packages
#install.packages("dendextend")
#install.packages("circlize")

# load package cluster
library(cluster, quietly = TRUE)
library(readxl)
library(dendextend)

##
## ---------------------
## Welcome to dendextend version 1.14.0
## Type citation('dendextend') for how to cite the package.
##
```

```
## Type browseVignettes(package = 'dendextend') for the package vignette.
## The github page is: https://github.com/talgalili/dendextend/
##
## Suggestions and bug-reports can be submitted at:
https://github.com/talgalili/dendextend/issues
## Or contact: <tal.galili@gmail.com>
##
##  To suppress this message use:
suppressPackageStartupMessages(library(dendextend))
## ---------------------

##
## Attaching package: 'dendextend'

## The following object is masked from 'package:stats':
##
##     cutree

library(colorspace) # get nice colors
library(circlize)

## ========================================
## circlize version 0.4.10
## CRAN page: https://cran.r-project.org/package=circlize
## Github page: https://github.com/jokergoo/circlize
## Documentation: https://jokergoo.github.io/circlize_book/book/
##
## If you use it in published research, please cite:
## Gu, Z. circlize implements and enhances circular visualization
##   in R. Bioinformatics 2014.
##
## This message can be suppressed by:
##   suppressPackageStartupMessages(library(circlize))
## ========================================

#Reading excel file
wolfH_test <- read_excel("howlpredict_test.xlsx")
str(wolfH_test)

## tibble [20 x 16] (S3: tbl_df/tbl/data.frame)
##  $ Serial              : num [1:20] 60 61 62 63 64 65 66 98 99 105 ...
##  $ howl_3ID.Filename   : chr [1:20] "151107-001_CG2_B2" "151107-
001_CG2_B3." "151107-001_CG2_B4." "151107-001_CG2_B5." ...
##  $ Individaul.Name     : chr [1:20] "CG2.A2" "CG2.A2" "CG2.A2" "CG2.A2"
...
##  $ Combo               : chr [1:20] "151107-001_CG2_B2(CG2.A2)" "151107-
001_CG2_B3.(CG2.A2)" "151107-001_CG2_B4.(CG2.A2)" "151107-
001_CG2_B5.(CG2.A2)" ...
##  $ Mark                : chr [1:20] "Test" "Test" "Test" "Test" ...
##  $ howl_3ID.Individual : chr [1:20] "CG2" "CG2" "CG2" "CG2" ...
##  $ class               : chr [1:20] "CG2" "CG2" "CG2" "CG1" ...
```

```
##  $ posterior.Baramati18: num [1:20] 4.46e-08 2.60e-04 5.44e-05 1.30e-01
5.81e-01 ...
##  $ posterior.CG1       : num [1:20] 0.000247 0.00493 0.003111 0.555948
0.365873 ...
##  $ posterior.CG2       : num [1:20] 0.9998 0.9948 0.9968 0.3141 0.0534 ...
##  $ posterior.Gangewadi2: num [1:20] 7.30e-40 1.98e-30 2.54e-32 2.07e-21
4.70e-19 ...
##  $ posterior.Nanaj1    : num [1:20] 6.58e-23 2.50e-15 9.24e-17 2.92e-09
2.63e-07 ...
##  $ x.LD1               : num [1:20] 3.552 1.634 2.012 -0.181 -0.833 ...
##  $ x.LD2               : num [1:20] 0.66 0.375 0.374 -0.605 -0.844 ...
##  $ x.LD3               : num [1:20] 0.0836 0.741 0.6546 -0.1032 0.2119 ...
##  $ x.LD4               : num [1:20] -0.65 -0.329 -0.388 0.201 0.353 ...
```

```
wolfH_test.rn <- data.frame (wolfH_test$Combo, wolfH_test$x.LD1,
wolfH_test$x.LD2, row.names = TRUE )
wolfH_test.rn
```

```
##                                     wolfH_test.x.LD1 wolfH_test.x.LD2
## 151107-001_CG2_B2(CG2.A2)                 3.55201449       0.65997729
## 151107-001_CG2_B3.(CG2.A2)                1.63401119       0.37543646
## 151107-001_CG2_B4.(CG2.A2)                2.01192695       0.37409372
## 151107-001_CG2_B5.(CG2.A2)               -0.18104712      -0.60456753
## 151107-001_CG2_B6.(CG2.A2)               -0.83264429      -0.84447684
## 151107-001_CG2_B7.(CG2.A2)                0.06785637      -1.11010737
## 151107-001_CG2_B8.(CG2.A2)                0.61311473      -0.03442798
## 151220-002_Baramati17_A1(BMT.SA2)        -4.88238566      -2.46409107
## 151220-002_Baramati17_A2(BMT.SA2)        -5.97480456      -1.92728814
## 151220-002_Baramati18_E1(BMT.SA2)        -6.62459497      -1.43385890
## 151220-002_Baramati18_E3(BMT.SA2)        -2.19843603      -2.05439749
## 160428-000_NUmred1_B1(NU.A)              -7.25235940      -0.07533194
## 160428-000_NUmred1_B2(NU.A)              -2.30755590       0.89996226
## 160428-000_NUmred1_B4(NU.A)              -1.86089725      -0.31835833
## 160428-000_NUmred1_B5(NU.A)              -7.29307956       0.75013502
## 160428-000_NUmred1_B7(NU.A)              -4.05030115      -1.18035646
## 151219-001_Baramati1_A1(BMT.A)            1.90699898       0.66161915
## 151219-001_Baramati1_A2(BMT.A)           -6.68595192      -3.53843013
## 151219-001_BaramatiX_A4(BMT.A)          -10.49370818      -4.33228431
## 151219-001_BaramatiX_A5(BMT.A)           -6.23042132      -1.50747248
```

```
str(wolfH_test.rn)
```

```
## 'data.frame':    20 obs. of  2 variables:
##  $ wolfH_test.x.LD1: num  3.552 1.634 2.012 -0.181 -0.833 ...
##  $ wolfH_test.x.LD2: num  0.66 0.375 0.374 -0.605 -0.844 ...
```

```
agn2 <- agnes(wolfH_test.rn, metric = "manhattan", stand = TRUE)

dend <- as.dendrogram(agn2)
# order it the closest we can to the order of the observations:
dend <- rotate(dend, 1:0)
```

```
## Warning in weights_for_order[order_x[order]] <- weights: number of items
to
## replace is not a multiple of replacement length
```

```
# Color the branches based on the hight:
dend <- color_branches(dend, h=2.2) #, groupLabels=different Howling
Individual)


# reduce the size of the labels:
# dend <- assign_values_to_leaves_nodePar(dend, 0.5, "lab.cex")
dend2 <- set(dend, "labels_cex", .4)
# And plot:
par(mar=c(1,1,1,1),  mgp=c(1, 1, 1))
circlize_dendrogram(dend2, lebels=TRUE, row.names= TRUE, labels_track_height
= 0.4)
```

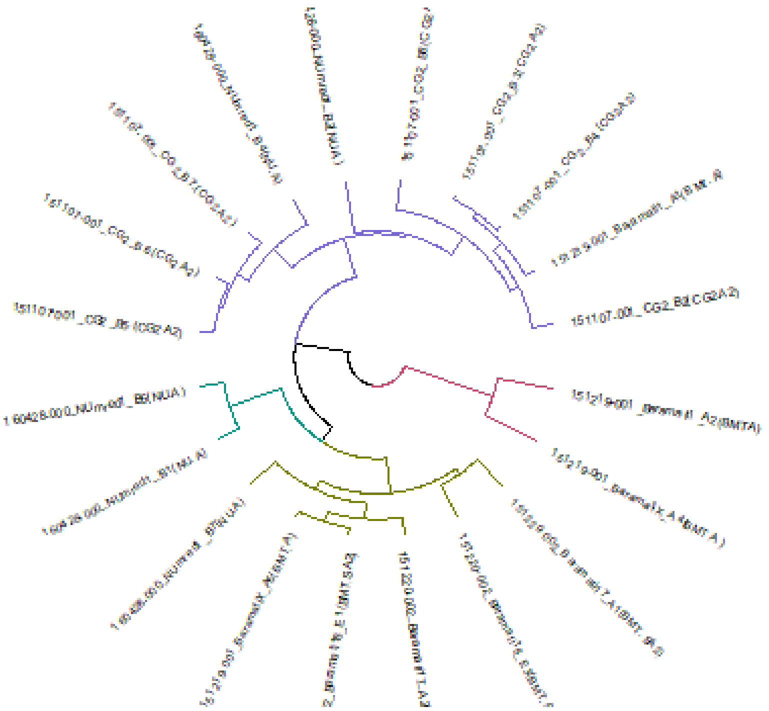

```
#writetable
clustnumber<- cutree(dend, h=2.2)
dendogram_test_howl <-data.frame(wolfH_test$Individaul.Name, clustnumber)
write.csv(dendogram_test_howl, "testhowl_dend_results2.csv")


#####################################################

#highlighting the wrong rows (optional)
highlight<- row.names(wolfH_test.rn)[13:14]
```

```
highlight2 <- row.names(wolfH_test.rn)[16:17]
highlight3 <- row.names(wolfH_test.rn)[20]

dend <- color_labels(dend, labels = highlight , col = 2)
dend <- color_labels(dend, labels = highlight2 , col = 2)
dend <- color_labels(dend, labels = highlight3 , col = 2)
# reduce the size of the labels:
# dend <- assign_values_to_leaves_nodePar(dend, 0.5, "lab.cex")
dend2 <- set(dend, "labels_cex", .4)
# And plot:
par(mar=c(1,1,1,1),  mgp=c(1, 1, 1))
circlize_dendrogram(dend2, lebels=TRUE, row.names= TRUE, labels_track_height
= 0.4)
```

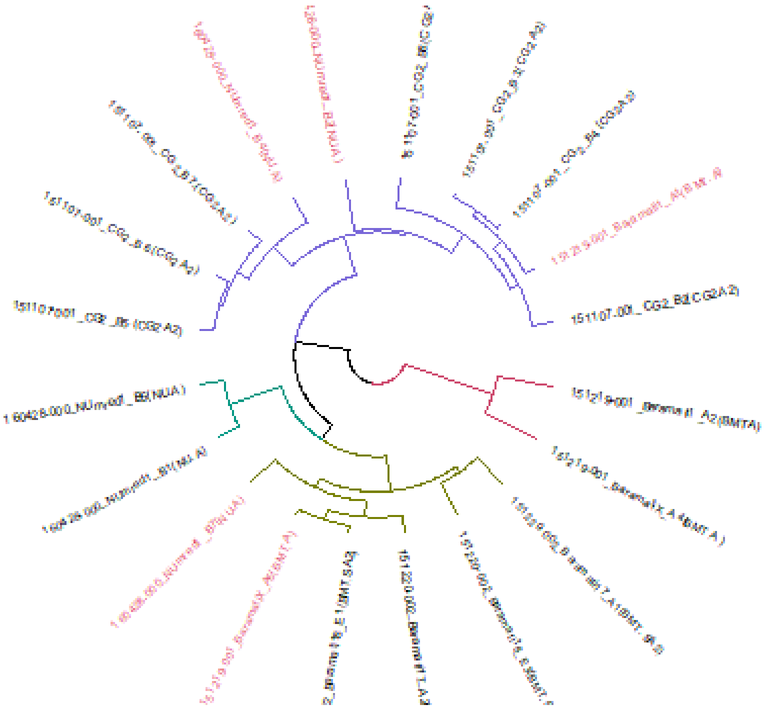

Supplement: Supplementary file 4 — Supplementary Information 4. [file 41598_2021_86718_MOESM4_ESM.pdf]
